# Supplementary material for: Observation of higher-order non-Hermitian skin effect
Source: Nat Commun. 2021 Sep 10;12:5377. doi: 10.1038/s41467-021-25716-y (PMC8433224; doi:10.1038/s41467-021-25716-y)
Supplement: Supplementary file 1 — Supplementary Information [file 41467_2021_25716_MOESM1_ESM.pdf]

Supplementary Information

Observation of higher-order non-Hermitian skin effect

Xiujuan Zhang[1], Yuan Tian[1], Jian-Hua Jiang[2,†], Ming-Hui Lu[1,3,†] and Yan-Feng Chen[1,3,†]

[1]*National Laboratory of Solid State Microstructures and Department of Materials Science and Engineering, Nanjing University, Nanjing 210093, China*

[2] *School of Physical Science and Technology, and Collaborative Innovation Center of Suzhou Nano Science and Technology, Soochow University, 1 Shizi Street, Suzhou 215006, China*

[3]*Collaborative Innovation Center of Advanced Microstructures, Nanjing University, Nanjing 210093, China*

[†]Correspondence and requests for materials should be addressed to joejhjiang@hotmail.com (JHJ), luminghui@nju.edu.cn (MHL) or yfchen@nju.edu.cn (YFC).

1. Theoretical model

In this section, we derive the theoretical model to characterize the designed sonic crystal (SC) and the observed non-Hermitian skin effect in the main text. Our SC is an acoustic analog of the spinful 2D Su-Schrieffer-Heeger (SSH) model, as shown in Supplementary Figure 1a, where the spin-up and spin-down modes are emulated by the anti-clockwise and clockwise resonant modes. For simplicity, here, we only study the model with clockwise modes. The case with anti-clockwise modes can be analyzed following the same derivations. When the clockwise modes are considered, the biased loss in Fig. 1a of the main text can be modeled by the anisotropic intra-cell coupling strengths, i.e., t_1 and $t_1+\chi$. The inter-cell coupling strengths are isotropic, denoted as t_2. The Hamiltonian in momentum space for such an SSH-like lattice can be written as

$$H=\begin{pmatrix} 0 & t_1+t_2e^{ik_x} & t_1+\chi+t_2e^{-ik_y} & 0 \\ t_1+\chi+t_2e^{-ik_x} & 0 & 0 & t_1+\chi+t_2e^{-ik_y} \\ t_1+t_2e^{ik_y} & 0 & 0 & t_1+t_2e^{ik_x} \\ 0 & t_1+t_2e^{ik_y} & t_1+\chi+t_2e^{-ik_x} & 0 \end{pmatrix}. \quad \text{(S1)}$$

Here, on-site energy is taken as zero and only the nearest-neighbor coupling is considered. The eigen-values and eigen-vectors of Eq. (S1) give the dispersions and wavefunctions of the lattice, respectively. In the Hermitian regime ($\chi = 0$), the staggered coupling parameters, i.e., $t_1 \neq t_2$, open band gaps, as demonstrated in Supplementary Figure 1b, where the energy dispersions for three cases, $t_1 = 3, t_2 = 1$, $t_1 = t_2 = 1$, and $t_1 = 1, t_2 = 3$, are successively presented. These dispersions are similar to those shown in Fig. 1b of the main text, suggesting that our system can indeed be described by the 2D SSH-like tight-binding lattice. Note that in Supplementary Figure 1b, the middle two energy bands are degenerate along the high symmetric line ΓM, while in Fig. 1b of the main text, such a degeneracy is lifted. This discrepancy is presumably due to the spin-degeneracy (i.e., the interferences between the clockwise and anti-clockwise modes) and the weak next-nearest-neighbor couplings in the SC. Nevertheless, Supplementary Figure 1b still captures the typical dispersion features of the SC.

Based on the analyses in the main text, the staggered coupling parameters bring forward distinct bulk topology, which is accompanied with parity inversion, as demonstrated in Fig. 1b. Such a parity inversion leads to non-trivial bulk polarizations. Here, we further calculate the bulk polarizations of the tight-binding lattice, which show consistency with the SC. In a 2D system, the bulk polarization is defined in terms of the Berry phase vector potential $\boldsymbol{\mathcal{A}}$, as

$$P_i = -\frac{1}{(2\pi)^2}\int_{BZ} d^2\mathbf{k}\,\mathrm{Tr}[\mathcal{A}_i]\,,\ i = x, y \tag{S2}$$

where $(\mathcal{A}_i)_{mn}(\mathbf{k}) = i\langle u_m(\mathbf{k})|\partial_{k_i}|u_n(\mathbf{k})\rangle$, with i denoting the directions of the two lattice vectors and m and n running over the bands below the considered band gap. $|u_{m,n}(\mathbf{k})\rangle$ is the periodic part of the wavefunctions of the mth (or nth) order band with wave vector $\mathbf{k}$. BZ refers to the Brillouin

zone. In the numerical calculation, where the Brillouin zone is discretized, Eq. (S2) can be further reduced to

$$P_i = \frac{1}{2\pi}\int_L \, d\nu_{i,k_j} \ , \ \ i = x, y, j = y, x \tag{S3}$$

Here, L denotes the projection length of the BZ along k_j direction. ν_{i,k_j} is the Berry phase along the loop k_i for a fixed k_j. Supplementary Figure 1c presents the calculated ν_x (ν_y=ν_x restricted by the inversion symmetry) for the two gapped cases in Supplementary Figure 1b (the lowest band is studied). Accordingly, the bulk polarizations are obtained as $\mathbf{P} = (0, 0)$ for $t_1 > t_2$ and $\mathbf{P} = \left(\frac{1}{2}, \frac{1}{2}\right)$ for $t_1 < t_2$, corresponding to the topological trivial and non-trivial cases, respectively. This is consistent with the parity inversion analyses in the main text.

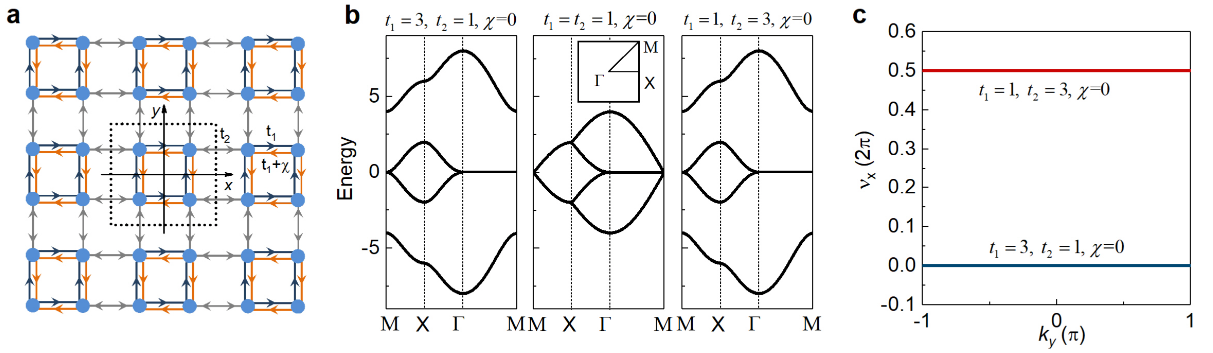

Supplementary Figure 1 | Theoretical tight-binding model. a, The tight-binding lattice that models the SC proposed in the main text. The dotted box encloses a unit-cell. **b,** Energy dispersions of the lattice in **a**, with coupling parameters taken as $t_1 = 3, t_2 = 1$, $t_1 = t_2 = 1$, and $t_1 = 1, t_2 = 3$, successively from left to right. The common parameter $\chi = 0$. **c,** Calculated Berry phase ν_x as functions of k_y for the two gapped cases in **b**. The bulk polarization is accordingly obtained by integration of ν_x over k_y.

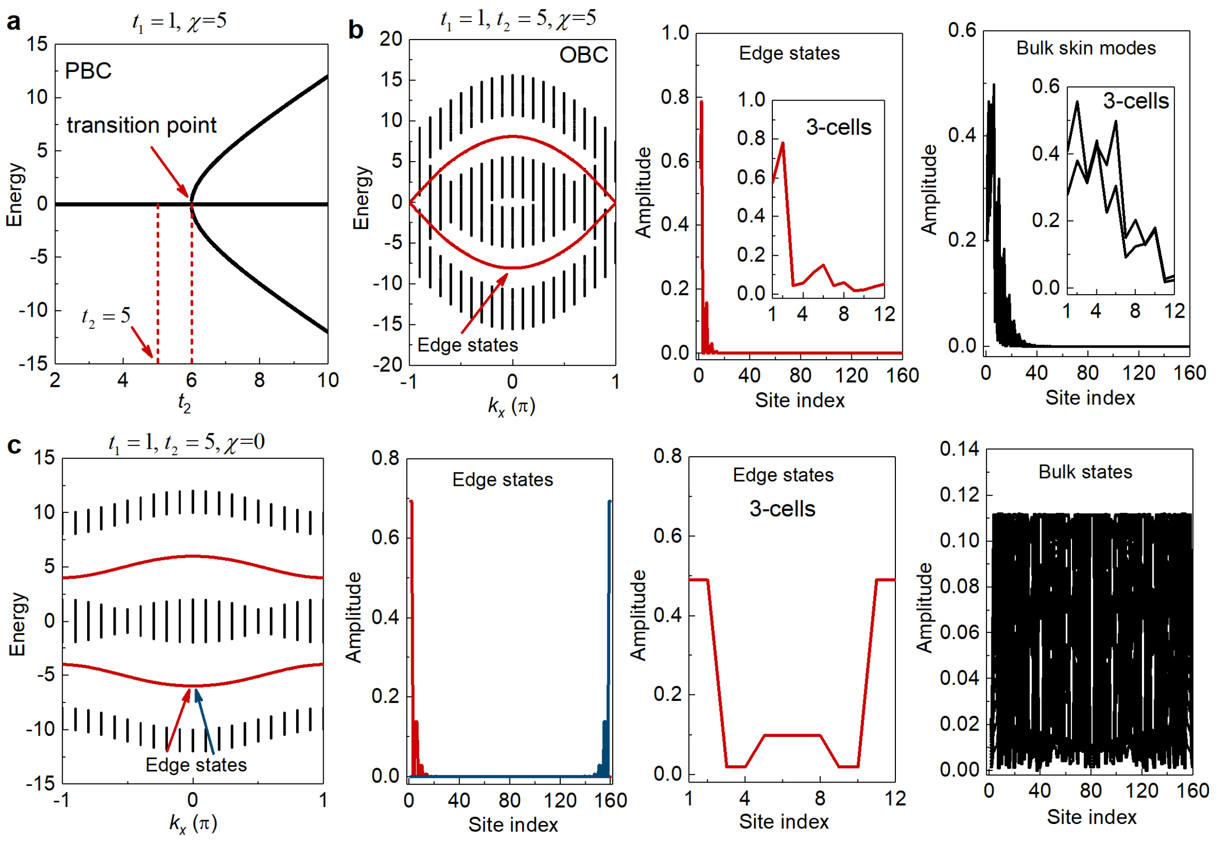

Supplementary Figure 2 | Non-Hermitian skin effect in the tight-binding model. a, The topological phase diagram under the PBC, with $t_1 = 1$ and $\chi = 5$ indicating a non-Hermitian system. **b,** Energy dispersions of a supercell with 40 unit-cells, subject to the OBC along the y direction and PBC along the x direction. Edge states are found, indicating a topological non-trivial phase at $t_2 = 5$, which shows an inconsistency with the prediction under PBC. The wavefunctions of the edge states (the middle panel) and the bulk states (the right panel) show wave localization toward the upper boundaries (with smaller site indices), as manifestations of the non-Hermitian skin effect. The results for the supercell with 3 unit-cells are also presented. c, The same as b, but with $\chi = 0$ indicating a Hermitian case. The wavefunctions of the edge states are localized around both the upper and lower boundaries. Meanwhile, the bulk states are no longer localized but become extended.

When non-Hermiticity is introduced, i.e., $\chi \neq 0$, the conventional bulk-boundary correspondence breaks down, signified by the different energy spectra between the lattices with periodic boundary condition (PBC) and with open boundary condition (OBC). In the main text, we numerically show this difference in Fig. 1c. Here, we study a specific case with $\chi = 5$ for an additional illustration. Supplementary Figure 2a presents the eigen-energy (the real parts) for the four eigen-states at the M point subject to the PBC as a function of t_2, where $t_1 = 1$ and $\chi = 5$ are fixed. It is seen that for a non-zero χ, the topological phase transition takes place at $t_1 + \chi = 6$, where the degeneracy of the upper and lower bands is lifted and band gaps are opened. Surprisingly, if we take $t_2 = 5$ (corresponding to a gapless phase under PBC) to compute the dispersions of a supercell subject to OBC along the y direction and PBC along the x direction, as shown in Supplementary Figure 2b (left panel), we find that edge states emerge (the red curves), indicating a topological non-trivial phase. Such an inconsistent topological phase transition subject to PBC and OBC suggests a non-Hermitian skin effect. As shown by the wavefunctions in the middle panel for the edge states (i.e., the two degenerate edge states with negative eigen-energy at $k_x = 0$, indicated by the red arrow) and in the right panel for the bulk states (i.e., the lowest 39 states at $k_x = 0$), indeed all the states are localized toward the upper boundary (with smaller site indices). Here, 40 unit-cells are taken in the calculations. We also present the wavefunctions for a case with 3 unit-cells (see the insets), which also show the interesting localization effects, for both the edge states and the skin modes. For comparison, the same studies are conducted in the Hermitian case with $\chi = 0$. The results are presented in Supplementary Figure 2c, which exhibit strikingly different behaviors from the non-Hermitian case. In particular, the edge states are no longer localized toward the upper boundary, but have preferences to both the upper and lower boundaries. Additionally, the bulk states become

extended. These results are consistent with those reported in the main text for the real metamaterial and provide additional evidence to the non-Hermitian skin effect.

2. Parity inversion at the high symmetric point X

In the main text, Fig. 1b presents the bulk topological transition, which is signaled by the parity inversion at the X point. To further confirm this, we provide the acoustic wavefunctions of the eigen-states on the lowest two bulk bands (at both the Γ and X points) below the concerned band gap in Supplementary Figure 3. It is shown that the parity at the X point indeed flips during the process of band gap closing and re-opening.

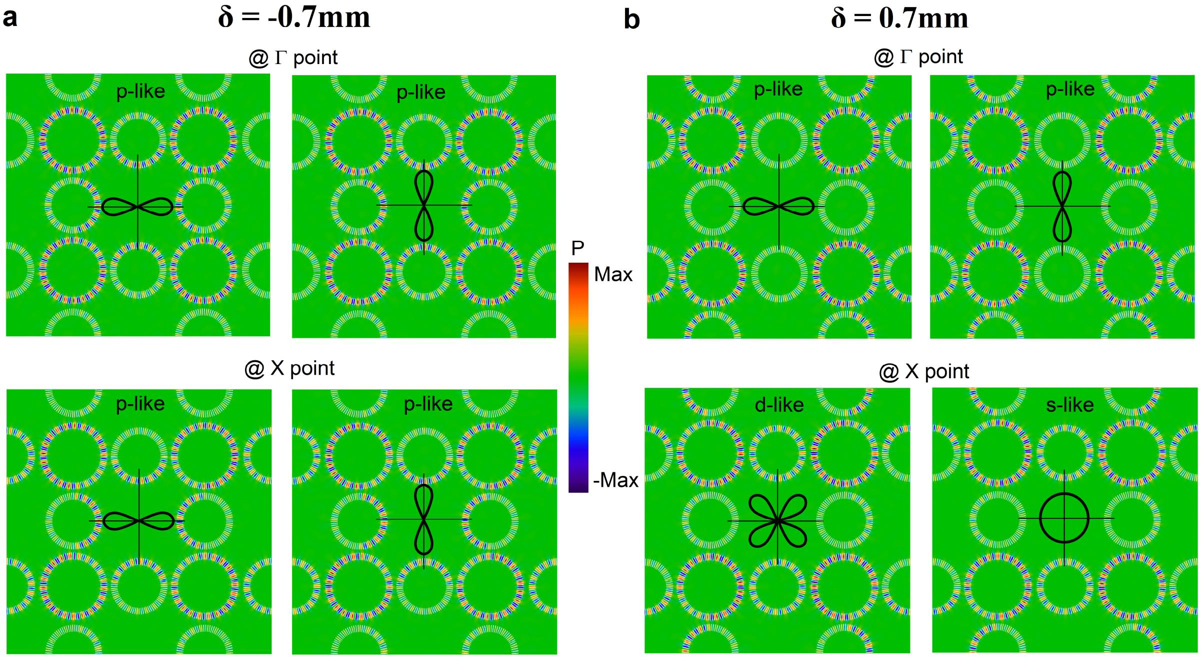

Supplementary Figure 3 | Parity properties of the bulk states at the Γ and X points. a, Acoustic wavefunctions for the four bulk states located below the band gap at the Γ and X points, for the case with $\delta = -0.7$ mm. It is observed that the states at both the Γ and X points have the p-like wave distributions. **b,** The same as **a**, only for the case with $\delta = 0.7$ mm. Note that now the

states at the X point become s-/d-like while their counterparts at the Γ point are still p-like, indicating a clear signature of parity inversion.

3. The degenerate topological edge states

Figure 2a of the main text presents the projected band structure of a ribbon-like supercell, which gives the in-gap edge states. We show here in Supplementary Figure 4 the frequencies of the eigen-states at $k_x = \frac{\pi}{a}$ as functions of the solution number and find that there are four nearly degenerate states emerging in the gap of the bulk states. Further simulations on the acoustic wavefunctions confirm that these degenerate states are indeed edge states.

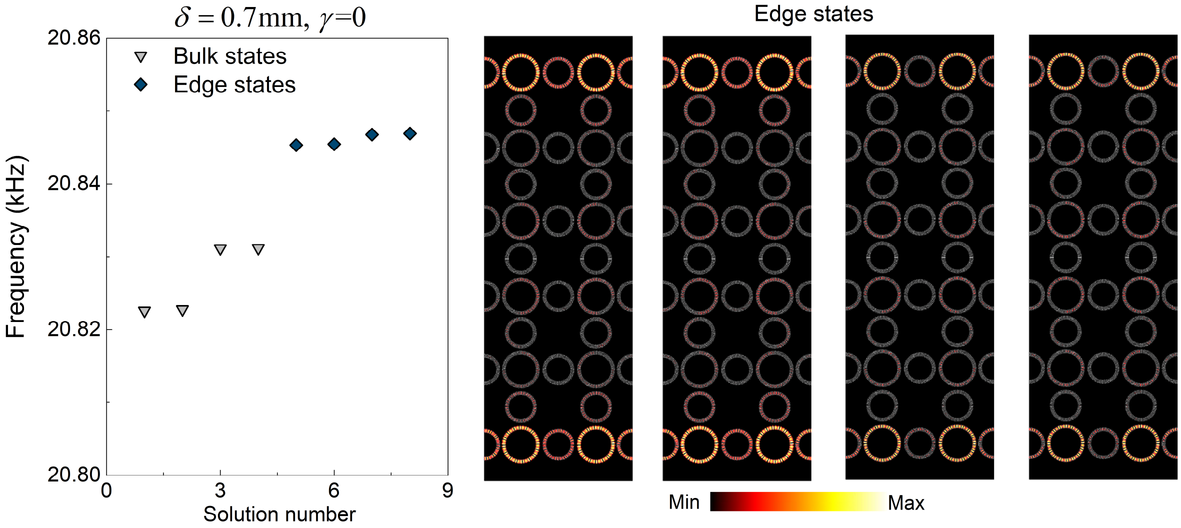

Supplementary Figure 4 | Degenerate topological edge states. Frequencies of the eigen-states at $k_x = \frac{\pi}{a}$, calculated for the ribbon-like supercell (the same as that in Fig. 2a of the main text), as functions of the solution number are plotted. Four in-gap, nearly degenerate states (dark blue) are observed. The corresponding acoustic wavefunctions indicate that they are topological edge states.

4. Sample photos and experimental set-ups

In this section, we illustrate the fabricated sample photos and elaborate the experimental set-ups for the measurements in Figs. 2-3 of the main text. Two samples are fabricated, including an edge sample and a corner sample. The former consists of 12 by 11 ring resonators (i.e., 3 by 3 unit-cells), with open boundaries along the *y* direction (see Supplementary Figure 5a for the sample photo). The latter consists of 7 by 11 ring resonators (i.e., 2 by 3 unit-cells), with open boundaries in both the *x* and *y* directions (see Supplementary Figure 5b for the sample photo). An enlarged image of the site and link rings is also shown. To induce non-Hermiticity, dissipative materials (i.e., the porous sponge) are added to the intra-cell link rings, forming a biased configuration, as illustrated in Supplementary Figure 5c for one unit-cell.

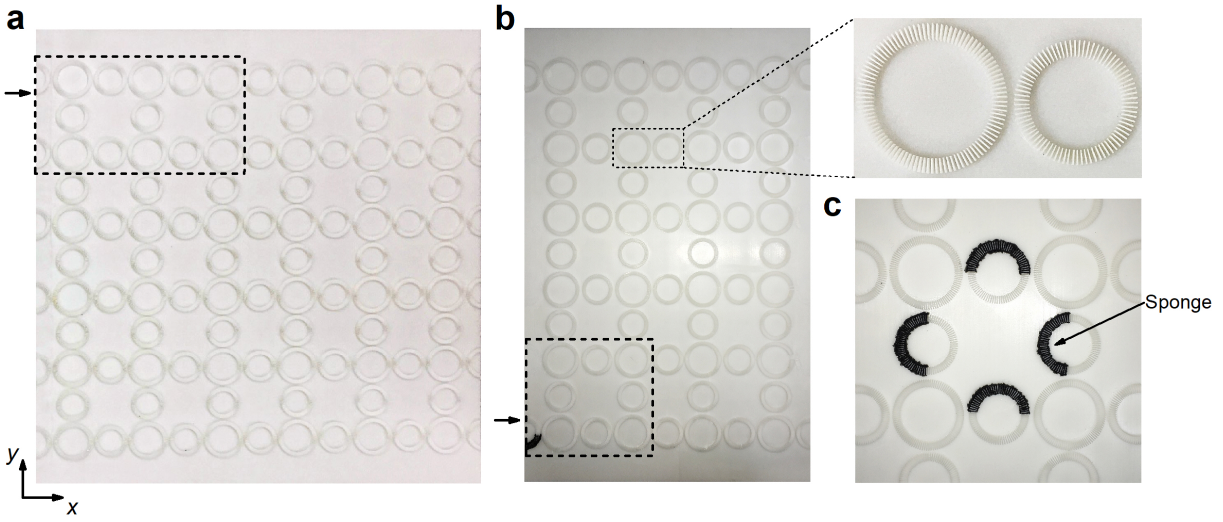

Supplementary Figure 5 | Fabricated samples. a, The edge sample with 12 by 11 ring resonators. **b,** The corner sample with 7 by 11 ring resonators. An image of the enlarged site and link rings is inserted. The thick dotted boxes in **a** and **b** mark the scanning domains in the measurements and the arrows indicate the input plane-wave-like signals. **c,** The biased loss configuration, formed by adding porous sponge to the air slits of the intra-cell link rings. Note that for clarification, here we

only show the loss domains in one unit-cell (in experiments, every unit-cell has the same loss configuration).

To conduct the measurements, an acoustically rigid plate is placed on top of the samples to form a waveguide. The plate is perforated with open channels in order to probe the acoustic fields. For each ring, there are 18 open channels forming the same ring-shape as the resonators (see Supplementary Figure 6a). During measurements, the detector is manually moved in a channel-by-channel fashion to scan the concerned domains. When one channel is probed, all the other channels are sealed by acoustically rigid plugs to avoid sound leakage (see Supplementary Figure 6a, lower panel). The obtained data are post-processed to generate the pump-probe transmission spectra and the acoustic field maps, as shown in Fig. 2c-d and Fig. 3c-e of the main text. It is pointed out that the transmission spectra for the bulk and edge probes in the edge samples (with and without biased loss) are obtained respectively by averaging the measurements on Rings 1-5 in the bulk region and by averaging Rings 6-9 on the edge. These ring indices are marked in Supplementary Figure 6b. For the corner samples (with and without biased loss), the transmission spectra for the bulk, edge and corner probes are respectively the averaging over Ring 1, 2 and 3 (the indices are marked in Supplementary Figure 6c).

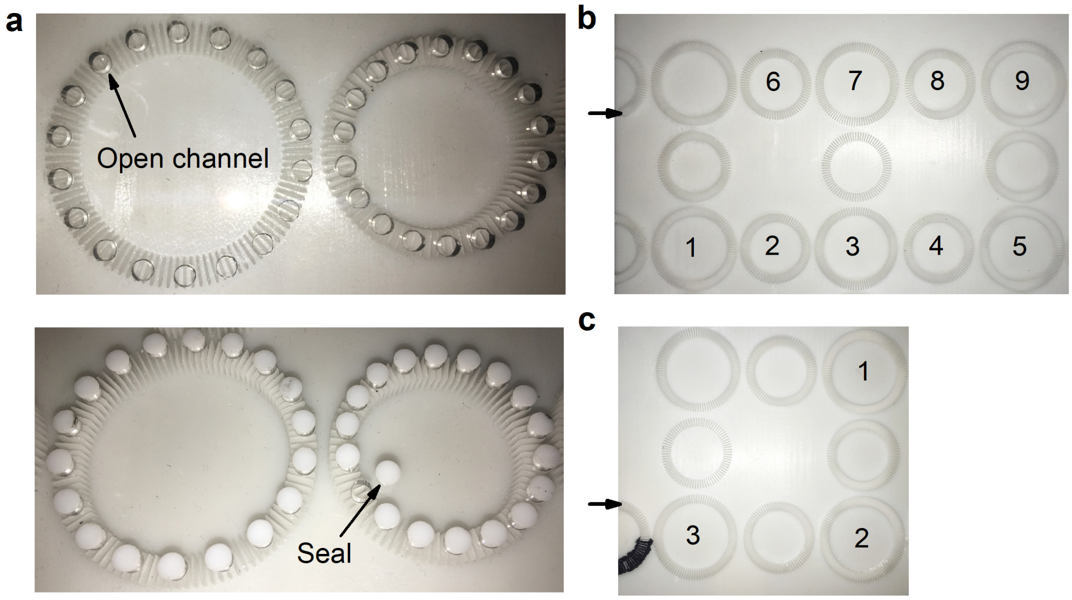

Supplementary Figure 6 | Measurement set-ups. a, Images of a partial upper board with open channels (covering the regions of a site ring and a link ring), as indicated by the upper panel. For each ring, there are 18 channels. During the measurements, the channels are either opened for detection or sealed to avoid sound leakage (see the lower panel for the seals). **b and c,** Respectively the scanned domains for the edge and corner samples, where the transmission spectra are obtained by averaging over the marked rings.

5. Robustness study of the non-Hermitian skin effect

In this section, we conduct systematical study on the robustness of the non-Hermitian skin effect. Essentially, the non-Hermitian skin modes emerge due to the interference effect of the clockwise (anti-clockwise) resonant modes driven by the non-Hermiticity, as schematically illustrated in Fig. 1d-e in the main text. They are not protected by the crystalline symmetry (this is different from the topological edge/corner states that are protected by the crystalline symmetry). Inducing perturbations indeed affects the skin modes to certain extent. Here, we conduct systematical

simulations to investigate the effect of perturbations of geometries and lossy factors on the skin modes.

The unperturbed case is presented for comparison, as shown in Supplementary Figure 7a. Three types of geometric perturbations are considered, including the perturbations on geometric corners, edges and bulks. We find that the skin modes are more sensitive to the perturbations on geometric corners and edges, compared with the bulk perturbations. This is due to the fact that the non-Hermiticity drives the resonant modes accumulating towards the edges and then towards the corners. If the edges and corners are perturbed, the interference process is sabotaged, accordingly affecting the formation of the skin modes. On the other hand, the resonant modes in the bulk can always re-route when encountering with perturbations, which therefore impose relatively small effect on the skin modes. For example, if we perturb the upper-right corner of the box-shaped sonic crystal structure (which has the same geometry as that presented in Fig. 3 of the main text) by shrinking the ring resonator by 0.1% (the perturbation is highlighted in the geometry, as shown by the left panel of Supplementary Figure 7b), the original topological corner states at the upper-right corner still exist, with the resonant frequencies shifted by ~0.07% (see the blue circles marked in the eigen-spectra, the middle panel of Supplementary Figure 7b). Note that in our system, the resonant frequencies of the ring resonators are rather sensitive to the ring diameters. Here, in order to clearly track the evolution of the skin modes, the geometric deformations are chosen to be small. We find that some of the skin modes are perturbed. Firstly, the original spin-down skin modes localized at the upper-right corner (as shown in Supplementary Figure 7a) are dimed or disappear, while the spin-up skin modes localized at the lower-left corner remain intact. This can be observed by the field distributions of three eigen-states, which are marked by orange arrows and numbers in the eigen-spectra (i.e., States 4-6). Secondly, two localized modes at the upper-right corner are

no longer spin-polarized, i.e., they contain both spin-up and spin-down components, forming standing wave patterns (as shown by the field distribution of State 7). The non-Hermitian skin effect is not manifested for these two corner states, exactly due to the lack of interference. Similar phenomenon is observed for other corner perturbations. For example, when the upper-left ring resonator is shrunken by 0.1%, the corresponding corner states with shifted frequencies do not exhibit skin effect, as shown by State 11 in Supplementary Figure 7c. In this case, however, because the upper-right corner is intact, the remaining states exhibit spin-polarized skin effect, similar to that in the unperturbed case, as exemplified by States 8-10.

In the case of edge perturbations, the non-Hermitian skin effect is again affected. As presented in Supplementary Figure 7d, where two site rings at the upper edge are shrunken by 0.1%, the interruption of the interference process at the upper edge leads to modification of some of the skin modes, as shown by the field distributions of States 12-15. In State 12, the spin-down skin modes at the upper-right corner disappear. In States 13-14, acoustic wave localization happens around the edge perturbations. For State 15, because the corner states at Hermitian limit couple to each other through evanescent waves which can re-route in the bulk region or along the other three intact edges, this skin mode is less affected by the upper edge perturbations.

Next, we consider bulk perturbations. As discussed above, the resonant modes can re-route when encountering with bulk perturbations. As a result, bulk perturbations impose small effect on the non-Hermitian skin effect. For demonstration, we present in Supplementary Figure 7e the eigen-spectra of the box-shaped structure with four site rings in the bulk region shrunken by 0.1%. It is seen that the total 32 states have almost the same eigen-frequencies as their counterparts in the unperturbed case. The field distributions of three selected skin modes (marked as States 16-18) also exhibit similar spin-polarized properties as the unperturbed skin modes.

When the designed loss domains are perturbed, the skin modes are affected depending on the perturbation strength. For example, we randomly select three link rings with loss (respectively in the bulk region, at the upper edge and near the lower-left corner) and deliberately set the loss factor to be $\gamma = 0.03$. The calculated eigen-spectra and the corresponding field distributions for three eigen-states are presented in Supplementary Figure 7f. It is seen that as long as the system is under the non-Hermitian condition, the skin modes remain negligibly affected. However, we find that if the perturbations on loss factor lead to break-down of non-Hermiticity (e.g., $\gamma = 0$), some of the skin modes can be severely disturbed. This demonstrates the important role of non-Hermiticity in the formation of skin modes.

Here, we remark that under perturbations of geometry and dissipation, only some of the skin modes in our system are considerably perturbed while the majority of the skin modes are negligibly affected. This demonstrates the robustness of the non-Hermitian skin effect against local perturbations.

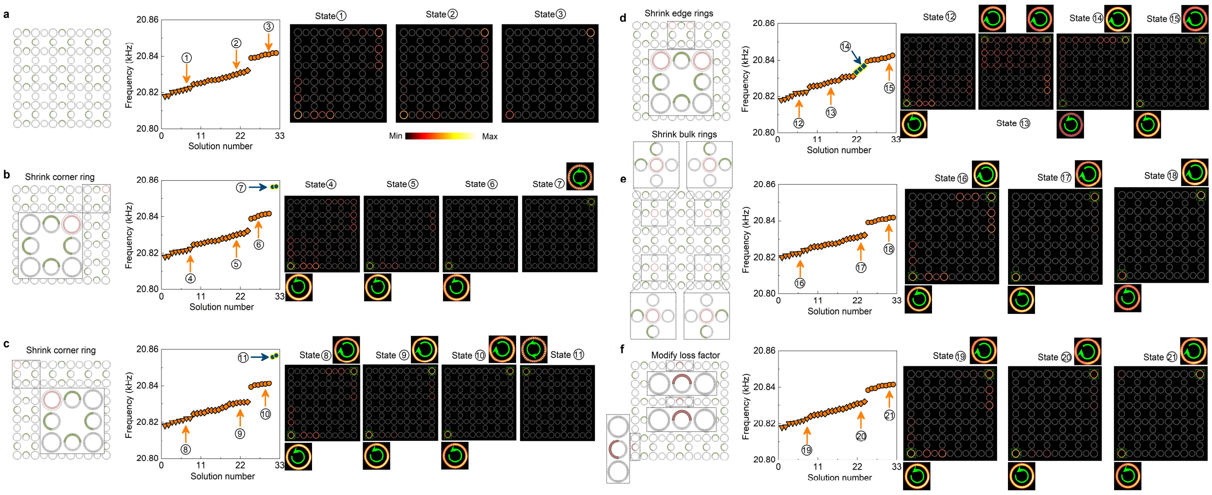

Supplementary Figure 7 | The effect of geometric and loss perturbations on the non-Hermitian skin modes. We present the eigen-spectra and field distributions to track the evolution

of the skin modes. Each sub-figure contains, from left to right, the geometric sketch of the box-shaped structure (with perturbations highlighted in red), the corresponding simulated eigen-spectra and acoustic field distributions for selected eigen-states (which are marked by arrows and labeled by numbers in the eigen-spectra). **a,** The case without perturbations, which is presented for comparison. **b-e,** Three types of geometric perturbations are considered, including the corner, edge and bulk perturbations. It is observed that the corner and edge perturbations have stronger effect on the skin modes, compared with the bulk perturbations. **f,** The perturbations on the loss factor. Three link rings in the bulk region, at the upper edge and around the lower-left corner are perturbed by setting the loss factor as $\gamma = 0.03$ in the simulations. It is shown that as long as the non-Hermitian condition is maintained, the skin modes are negligibly affected.

6. Non-Hermitian skin effect in trivial insulators

In this section, we show that the non-Hermitian skin effect does not necessarily require the material to be topologically non-trivial. We conduct simulations on the non-Hermitian skin effect in the trivial insulator with $\delta = -0.7\text{mm}$ and $\gamma = 0.01$ (see Supplementary Figure 8). It is seen that although the trivial insulator does not support topological edge/corner states in the bulk gap, it still exhibits the non-Hermitian skin effect. Similar to those presented in Figs. 2-3 for the higher-order topological insulator (HOTI), in the trivial insulator, the original bulk states at Hermitian limit become spin-polarized skin modes. Specifically, for the structure with PBC along the x direction and OBC along the y direction, the spin-up skin modes travel toward the lower boundary while the spin-down skin modes appear around the upper boundary (see Supplementary Figure 8a-b). For the structure with OBCs along both directions, the spin-up skin modes are now localized at the lower-left corner while the spin-down skin modes are accumulated at the upper-right corner (see Supplementary Figure 8c-d).

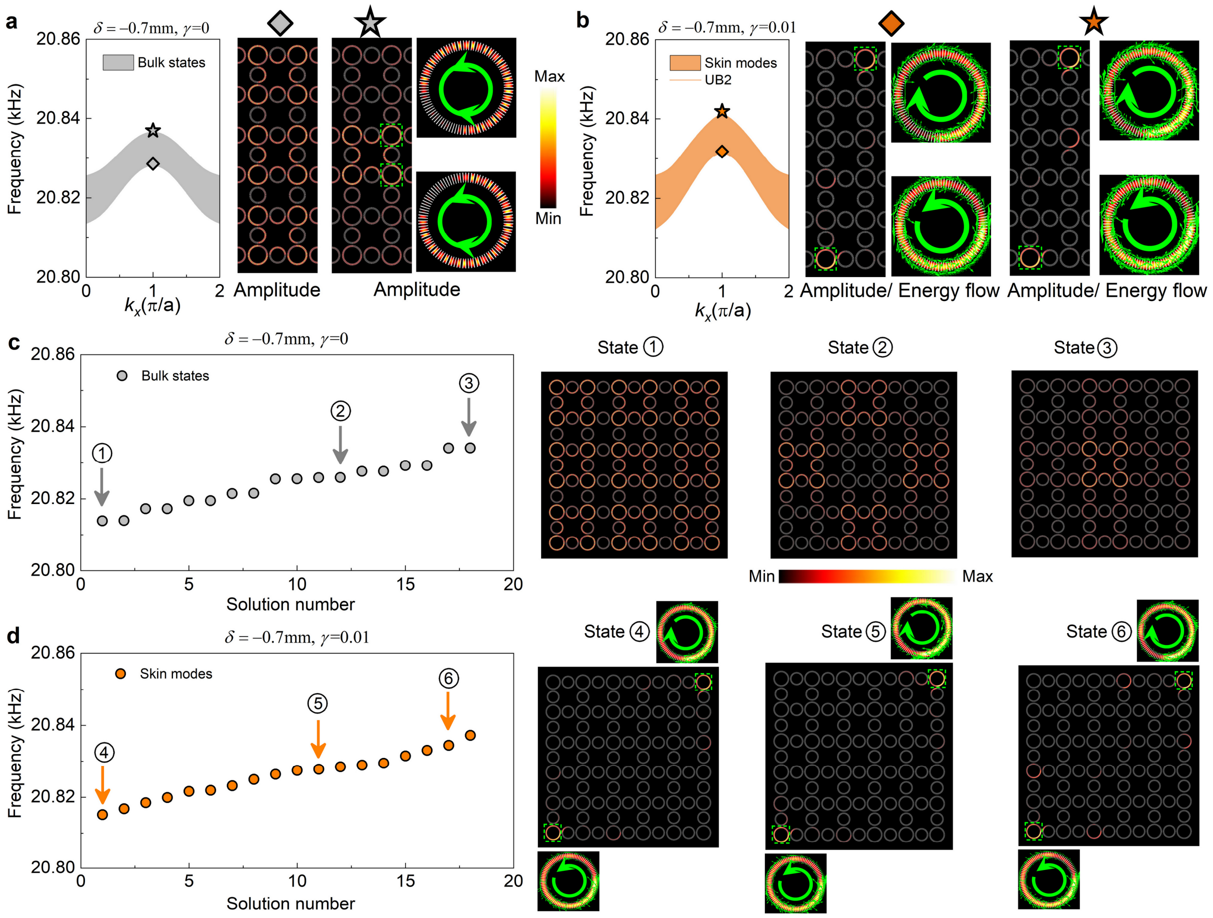

Supplementary Figure 8 | Non-Hermitian skin effect in the trivial insulator. a, Calculated eigen-spectrum for a supercell with PBC along the x direction and OBC along the y direction in the Hermitian regime. Note that in the trivial insulator, the topological edge states no longer emerge. Acoustic pressure field profiles for two marked states (grey diamond and star) are presented, showing the typical extended bulk states, whose mode patterns are standing waves due to the spin-degeneracy. **b,** The same as **a,** only with acoustic loss. Due to the non-Hermitian skin effect, the original bulk states become edge-like skin modes (as shown by the two marked states). Similar to that in the HOTI, the non-Hermitian skin effect is spin-polarized, with spin-up states accumulating toward the lower boundary while the spin-down states accumulating toward the upper boundary. **c and d,** Eigen-spectra for the box-shaped supercell (OBC in both the x and y

directions) without and with acoustic loss, respectively. Note that the absence of topological edge and corner states does not affect the spin-polarized non-Hermitian skin effect, which is again manifested when the acoustic loss is introduced. The spin-up skin modes reside near the lower-left corner while the spin-down modes are localized around the upper-right corner. This is consistent with that observed in the HOTI.

7. Broadband non-Hermitian skin effect

In this section, we argue that due to the existence of an extensive number of skin modes, the non-Hermitian skin effect is a broadband effect. As demonstrated in both the main text for the real metamaterial and the above Section 1 for the tight-binding model, when non-Hermiticity is introduced, all the states become skin modes. These states occupy finite frequency regimes and are experimentally detectable. In Supplementary Figure 9, we present the simulated transmission spectra for the edge and corner samples without ($\gamma = 0$) and with ($\gamma = 0.02$) the designed loss. The set-ups are the same as the experiments, where a plane wave source is injected into the samples and the numerical probes are conducted in the bulk, edge and corner regions (using the same averaging as described in Supplementary Figure 6b-c) to obtain the transmission spectra. The results indicate that the non-Hermitian skin effect is salient in broadband frequency regimes. The acoustic field maps at the same excitation frequencies as the experiments are also presented, which show high agreements with those presented in Figs. 2c-d and 3c-d of the main text, suggesting that our experimental results can indeed reflect the non-Hermitian skin effect.

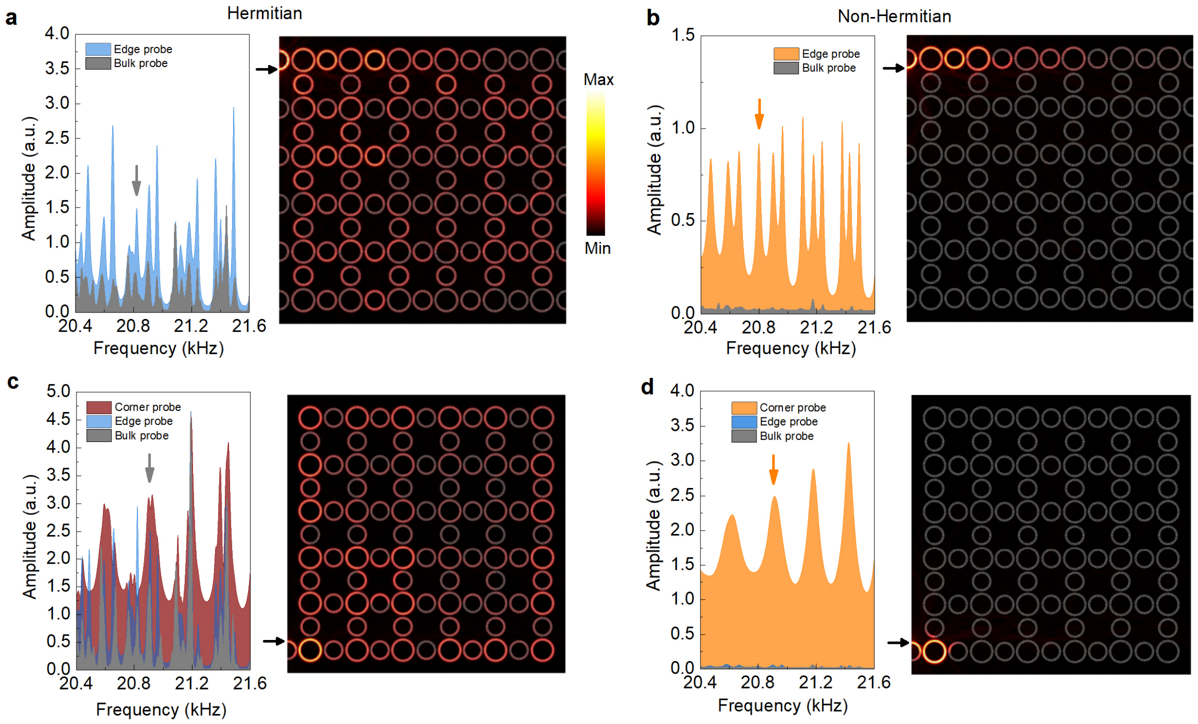

Supplementary Figure 9 | Simulated pump-probe transmission spectra. a and b, Respectively the same as that in Figs. 2c and 2d in the main text, but for simulations. It is clearly seen that the non-Hermitian skin effect exhibits a broadband behavior, consistent with the experimental measurements. Note that due to the background air loss, the peak spectral features in the simulations are flattened in the experiments. The acoustic field distributions for two excited states (with the same excitation frequencies as the experiments) also demonstrate the consistency with the experiments, i.e., after introducing the designed loss, all the states, including the bulk and edge states, become edge-like skin modes. **c and d,** Respectively the same as that in Figs. 3c and 3d in the main text, but for simulations. Again, clear features of the broadband higher-order non-Hermitian skin effect is observed when the extended states become localized corner-like skin modes after introducing the designed loss.
